# Pet owner and vet interactions: exploring the drivers of AMR

**DOI:** 10.1186/s13756-018-0341-1

**Published:** 2018-04-02

**Authors:** Matt Smith, Caroline King, Mark Davis, Adele Dickson, Jeni Park, Fraser Smith, Kay Currie, Paul Flowers

**Affiliations:** 1 Institute for Applied Health Research, Glasgow Caledonian University, Glasgow, Scotland G4 0BA UK; 2 School of Social Sciences, Monash University, Melbourne, 3800 Australia

**Keywords:** Antibiotic resistance, Pets, Vets, Behaviour, AMR

## Abstract

**Background:**

Antimicrobial resistance (AMR) is a growing public health problem across the world. As the negative consequences of AMR become apparent at local, national and international levels, more attention is being focussed on the variety of mechanisms by which AMR is potentiated. We explore how interactions between pet owners and veterinarians represent a key arena in which AMR-related behaviours can be shaped.

**Methods:**

In depth semi-structured interviews were carried out with pet owners (*n* = 23) and vets (*n* = 16) across the UK in 2017. A thematic analysis approach was taken, with inductively gathered data analysed deductively using a behavioural framework to identified key behaviours emerging from participant accounts which were amenable to change.

**Results:**

Interactions between vets and pet owners were characterised by misunderstandings and misconceptions around antibiotics by pet owners, and a lack of clarity about the positions and intentions of the other party. Vets and pet owners had differing perceptions of where pressure to prescribe antibiotics inappropriately originated. Vets perceived it was mostly pet owners who pushed for inappropriate antibiotics, whereas pet owners reported they felt it was vets that overprescribed. Low levels of understanding of AMR in general were apparent amongst pet owners and understandings with regard to AMR in pets specifically were almost non-existent in the sample.

**Conclusions:**

Improved use of antibiotics could be assisted by educating the pet owning public and by guideline development for companion animal vets, concurrent development of mandatory legislation, increased consultation time to facilitate better communication, development of vet training on antimicrobial therapy and stewardship led interactions with pet owners, and increased levels of knowledge of pet-related AMR amongst pet owners.

## Background

Interdisciplinary consensus identifies AMR as an increasingly severe global concern [1, 2]. Antibiotics have facilitated revolutionary improvements in medical practice in many areas (from routine to critical care) by protecting patients from the threat of bacterial infection [1]. However, due to various biological mechanisms (dependent on the infective organism and class of antimicrobial agent), there are now an increasing variety of microbes that display resistance to antimicrobial drugs [3–5]. The process that drives this resistance is selective pressure (causing differential mortality and growth stasis of non-resistant organisms which then causes genetic change in the overall population) of antimicrobial use, resulting in successive generational increases in resistance to mortality in bacteria [6]. Antimicrobial resistance (AMR) threatens to undo decades of medical progress, resulting in substantial increases of dangerous infections in the course of previously routine treatment [7, 8]. Resistant organisms are linked to increased disease severity due to lessened treatment efficacy, longer hospitalisation and increased costs in treatment and post disease care [7].

Animal health care is implicated in the problem of AMR. Animals have been shown to have potential to act as reservoirs for resistant organisms, especially when infections are not comprehensively and successfully treated. Moreover, veterinary science acknowledges that a unique and critical aspect of AMR in pets is their close physical contact with humans [9, 10]. This can lead to an increased potential for transmission of resistant microorganisms between humans and their pets [11], especially those that reside on the skin or in saliva [12]. In addition, pet numbers have substantially increased in the modern age, and more efforts are devoted to pet welfare, meaning higher levels of treatment for sick animals, and more frequent use of antibiotics for pets [9]. This is of particular concern for antibiotics also used in human medicine, especially those known as “last resort” treatments for potentially fatal infections [10]. Growing resistance to these antibiotics in particular is likely to result in increased human mortality [10]. Interspecies transmission can result in an increase of AMR through a feedback loop of resistance reservoirs and evolving generations of increasingly resistant bacteria [13–15].

To combat these drivers of AMR, the effective use of antibiotics in animal health depends on pet-owners and their collaborations with prescribing vets. Pet owners mediate the treatment of their animals; they control antibiotic usage and other actions that can directly determine potential for AMR in their animals [14]. In the absence of more effective antimicrobials as a solution to AMR, understanding the behaviours of pet owners in collaboration with vets could become an important part of the global effort to reduce AMR. Responsible use of antibiotics, or “Antimicrobial Stewardship” [16] will be referred to in this paper. Fishman [16] defines it as directly related to prescribing practices that ensure that antibiotics will continue to be effective for future generations. Here we conceive of it more broadly, as also taking into account responsible use by patients and pet owners to the same ends. In what follows, we investigate how pet-owners and vets talk about animal health care, antibiotics and antimicrobial resistance to advance understanding of AMR-related behaviour and opportunities for change.

### Study aims

This paper addresses the pet-owner – vet prescribing interaction through qualitative interviews focussed on experiences and knowledge of antibiotics and AMR, to i) shed light on pet-owners and vets experiences, beliefs and intentions with regard to their interactions on antibiotic prescription, and ii) to identify approaches for behaviour change interventions targeting pet owner-vet interactions as a primary site for mutual understanding between parties to develop more responsible antibiotic use in pets.

### Research questions

1. What do vets believe are the most important aspects of interaction between pet owners and themselves that can lead to increases in AMR?
2. What do the pet owning public believe are the most important aspects of interactions between themselves and vets that can lead to increases in AMR?
3. How do these aspects help or hinder the development and maintenance of antimicrobial stewardship?

## Methods

We took a qualitative approach, using semi-structured interviews with two types of study group: pet owners (*n* = 22) and small animal vets (*n* = 16) across the UK in 2017.

### Ethical approval and recruitment

Ethical approval for the study was granted from the ethics committee for Nursing and Community Health at Glasgow Caledonian University (Reference number: HLS/NCH/16/001).

Pet owners were recruited through: adverts on social media (Facebook and Twitter); convenience sampling at a veterinary practice; snowballing via recruited participants; and through personal contacts from the research team. Inclusion criteria for pet owners were: having a dog, cat or rabbit; having received antibiotics for their pet in the last year; and not having recently bereaved of a pet. Vets were recruited through: nationwide veterinary practices, networks and connections from Health Protection Scotland (HPS) and the wider Control of Antimicrobial Resistance Scotland (CARS) programme Animal Health and AMR group. The vet inclusion criterion was that they had to be in small animal practice.

### Data collection

Topic guides for interviews were developed with expert advice from the study advisory group. Both topic guides were used to balance participant led data with our focus upon understandings and perceptions of AMR and the drivers of AMR.

Twenty one pet owner participants were interviewed of which 14 were female and seven were male. For pet owners, eight face to face interviews and 13 phone interviews were carried out. The duration of the interviews ranged from 28 min to one hour and 30 min. Pet owners ranged in age from 32 to 77 years old.

Sixteen vets took part in interviews. Nine of the participants were male and seven were female. Eleven of the vets were based in Scotland, four in England and one in Northern Ireland. Recruited participants were identified as holding various positions within the industry including: front-line (*n* = 10), Second opinion (*n* = 2), vet hospital (*n* = 1), emergency out of hours (*n* = 1) and vet school (*n* = 1). These interviews lasted between 25 min and 40 min.

### Data analysis

Data were analysed thematically, as defined by Braun & Clarke [17], in two main stages. Initially three researchers independently coded a selection of transcripts looking for an agreed range of broad inductively developed descriptive themes, generating a list of codes. These broad codes were applied to all the remaining data using NVivo qualitative analysis software [18]. Coding was quality checked as an ongoing process by multiple researchers within the team.

Subsequently, within the second stage of analysis, the broad inductively derived data were analysed deductively using a behavioural analytic approach. Data relating to specific behavioural domains were identified amongst both samples. For vets, these included the following behavioural domains: prescribing; use of diagnostic testing; and interactions with pet owners. For pet owners, these included: appropriate use of antibiotics; AMR transmission related behaviours (between owner and pet); and interacting with vets. This paper examines the behavioural domain of interactions from the perspectives of both groups of participants. A continuous process of cross checking was carried out between researchers to ensure analytical rigour.

## Results

The following sections consider important AMR-related aspects of the broad behavioural domain of interactions between vets and pet owners, firstly from the perspective of vets, then from the perspective of pet owners.

### Vet perspectives on AMR-related interactions with pet owners

Vets gave their perspectives on their interactions with pet owners, and identified the behaviours, and barriers and facilitators they thought contributed to inappropriate antibiotic decision-making. These relate primarily to perceived pressures from pet owners and possible discussions around antimicrobial resistance.

### Perceived pressures from pet owners that influence antibiotic decision making

Vets reported that their interactions with pet owners were complex when it came to their decision making concerning antibiotics. There was a common tension between an awareness of the need for AMR stewardship and the perceived satisfaction of the pet owner. The satisfaction of the pet owner, from a vet perspective, was dependent on a myriad of factors which extended beyond the appropriate use of an antibiotic relating to clinical need, including: payment for consultation and the expectation of active treatment; ease of administering the treatment; previous experiences with receiving antibiotics for their pets; and pet owners’ emotional connections with their animals. Vets commonly reported prescribing ‘just in case’ in response to their perceptions of client anxiety about pet health and welfare. From the vet’s point of view, clients appeared to expect the provision of a clearly defined treatment (i.e. an antibiotic prescription), as the following example indicates:

There's a pressure when an owner comes in and pays 35 or 40 pounds , for a consultation, because their animal is unwell, that they want to go home with a medication that in inverted commas “fixes” their animal, and even though the bill will be higher with that medication, so you sort of have that feeling that if you have an unwell animal and if you send it home with nothing then you'll get a raised eyebrow, or for example they will say to you, “Well, so and so , the senior partner gave my older dog antibiotics just last week for similar symptoms” so, you know whatever, but there is a sort of general expectation I think of owners, that you will treat their animal when they pay for a consultation. (Vet 10)

As this extract suggests, expectations from clients to prescribe antibiotics were often inferred rather than explicitly stated. Vets saw that pet owners appeared to perceive prescription as a binary of presence or absence of treatment and therefore action versus inaction. Prescription appeared to be received as a measurable, visible action taken for the health of the pet, whereas a lack of prescription meant nothing was being done to help the animal.

Tacit pressure was also framed by vets in terms of pet owners’ expectations for transaction, that is, payment exchanged for a tangible outcome in the form of medicine for the pet. This expectation was exacerbated by the fact that veterinarian practices are profit making enterprises, and seeking to keep customers loyal, sometimes against appropriate clinical action. This pressure was felt by vets because customers are able to go to other practices where they may get what they want:

Yeah, I mean these people are our customers and they are, what keeps the business going, so if we annoy them and there is another vet practice they can go to where they may just be handed out antibiotics, and [the vet] might take the opinion “well, here’s a client, if I give him a course of antibiotics, he’ll just take his dog to me from now on and I’ve gained a client” (Vet 1)

These market pressures can therefore be construed as potential drivers of inappropriate antibiotic prescribing among other mechanisms. Other vet accounts show that the AMR related consequences of over-prescription are not high on their agenda when acquiescing to pet owner pressure to provide treatment. Vets cited professional tendencies to prescribing, i.e., giving animals antibiotics as a quick and easy way to deal with an infection, even if that was not necessarily the best course of action:

So for example, if you’ve got a cat with cystitis and a lot of [vets] will give them antibiotics when actually a lot of them don't need antibiotics. So I think a lot of is through fear as well. You want to make the animal better, therefore you think oh sod it, I'll just give them a jag. (Vet 13)

As this quotation indicates, the risks involved in inappropriate use of an antibiotic were outweighed by the risk losing a client. Potential, ambiguous and non-present consequences of AMR for vets were not as salient as the present lived experiences of pressure from pet owners, and business pressures around client satisfaction.

### Discussion of AMR with pet owners that influence antibiotic decision making

Vets were asked to what extent they felt they could use the concept of AMR to negotiate with pet owners in the interactions described above, to improve overall antimicrobial stewardship. Vets identified that pet owner understanding and willingness to discuss antibiotics and AMR were important determinants of their eventual prescribing decisions. The majority of vets reported that their clients tended to have some knowledge of the importance of correct antibiotic use; particularly that partial use of a course of antibiotics, or using them unnecessarily, had some kind of negative effects concerning future treatment outcomes. However vets reported a general lack of knowledge of the wider and longer term, inter specifies effects of over- or mis-use of antibiotics, and an almost total lack of knowledge of about the phenomena of AMR. This was also borne out in data from pet owners. Vet accounts also reported that clients were often unaware of disease processes. The limits of pet owners’ knowledge about appropriate antibiotic use could lead them to believe that an antibiotic would be appropriate treatment when it would not be suitable, for example, for a viral infection:

They'll be a little bit annoyed that I haven't really given them anything, even though there's nothing that I could really give them that would improve their cat's position. And a lot of the time, they will sort of be like, well, what about some antibiotics, what about this, what about that, and I'll have to be, like, well, no, your cat doesn't need it and it's not going to help. But they sort of feel like I'm not really listening to their issue or that I'm not trying to help their cat because I'm not giving them this medicine that they perceive will be helpful to them, basically. (Vet 3)

Most vets in the sample perceived that whilst this lack of knowledge was an issue, awareness was improving, making it easier to carry out AMR negotiations without jeopardising pet owner satisfaction. However, some vets reported that there were still those who would not engage in discussion about appropriate use of antibiotics:

You can tell very quickly if someone’s going to be receptive to that [appropriateness of antibiotics] conversation or not, and if they’re not, then obviously sometimes you just have to accept that that’s what the client wants, and if we don’t do that then the animal is going to suffer anyway, so we just say, well, there’s no point even discussing this. (Vet 15)

This extract shows that vets are required to make judgements which balance outcomes for clients and pets, revealing prescribing decisions to be complexly embedded in management of the pet’s illness, the owner’s expectations and the reality of a competitive business environment. Antimicrobial stewardship does not easily factor into these complex deliberations.

Vets also reported that their professional experience played an important role in empowering them to be able to assert a more appropriate course of action, skills which took time to develop:

I think a lot of it boils down to communication, and it does get easier as you get older. As a younger vet - I'm kind of middle aged now, somewhere in the middle - as a younger vet, it's difficult, you often feel pressured from clients, you feel pressured to conform to what the older vets would do, if they would do something in a certain way. But now that I'm an established vet in my own right with my own ideas and opinions, as long as I get the time to actually communicate that with the client and talk effectively, I can usually talk them round to my point of view and they will see that's reasonable enough, yeah. And I think it depends where you work. If you're in a very busy practice or if you're a younger vet feeling pressure from clients or from senior staff, then it is going to be difficult, you're just going to grab the antibiotics, that's the easy option. (Vet 2)

This account reflects the social norms and dynamics which influence prescribing decisions and underlines that the development of communication skills was an important factor in being able to negate pressures from misinformed pet owners and, it seemed, other vets. Identifying and sharing these skills could be a useful tactic for the advancement of stewardship.

### Pet owner’s perspectives on AMR-related interactions with vets

Pet owners also gave their perspectives on their interactions with vets, and identified the behaviours, and barriers and facilitators they thought contributed to antibiotic decision-making and antibiotic use for their pets. These relate primarily to pet owner expectations around antibiotic prescribing and discussions related to antimicrobial resistance.

### Pet owners’ expectations of antibiotic use

In contrast to vet perspectives that pet owner satisfaction was the main source of inappropriate prescribing, pet owners, on the whole, denied that they would be dissatisfied if their pets were not prescribed antibiotics. The majority of our respondents indicated that they would follow the vet’s instructions because they perceived vets to have expertise in an area where they had little to no knowledge.

I would say ultimately it’s my decision but the vet is the expert… I mean if they tell me it’s needed, I’ll not, I’m not going to, I might try and ask sensible questions… but I’m not going to dispute that. (Laura, dog owner).

However, some participants reported that they thought it was vets who promoted antibiotics unnecessarily:

But I have been to the vet before and got antibiotics and questioned it and asked why they were taking them, when, where… they said right we’ll put them on antibiotics and I said I’m not really sure that they need them and do we really need to go ahead with them and they said to me, yes we really, really, need to go ahead, then I would go ahead with it. But there has been instances where I’ve said, I don’t, I’m not sure that we need them, are you absolutely sure? And they said OK, no. We’ll wait a while. (Andrea, dog owner).

Andrea’s account of questioning the prescription was rare in our interviews as most reported that they had not had any disagreements with vets over their expectations that antibiotics would be prescribed. Some pet owners reported assuming that antibiotics were required and had those assumptions negated by the expertise of the vet. However, in contrast to vet perceived risks of not keeping the client satisfied, there were no cases where pet owners had major disagreements which resulted in moving to other vets. These accounts indicate that pet owners may differ markedly from vets regarding their experiences of interactions and that there was a shared tendency among pet-owners and vets to externalise the drivers of inappropriate prescribing. This understandable self-protecting bias among both vets and owners is an important consideration for the development of effective stewardship, as we discuss below.

### Pet owners’ understanding of AMR

As with vets, being able to understand and discuss AMR was seen by pet owners as an important factor for their own enactment of stewardship principles.

The majority of pet owners reported that they did not know about AMR, particularly in relation to their pets, confirming the reports of vets described above. While most pet owners did not know about AMR, the majority of participants did have an awareness of “superbugs”, which they described as being primarily hospital-based resistant infections such as MRSA. This awareness was mainly credited to news media. Only a few spoke in specific terms about AMR, for example, the mechanisms through which it might develop and what impact improper use of antibiotics might have on its development. It was usually discussed in more general terms:

“I think we’re just reaping the [consequences] of maybe people having antibiotics unnecessarily.” (*Maura, dog and cat owner*).

Even fewer pet owners had an understanding that AMR was an issue for animals and a very small minority knew about interspecies transmission. When participants were prompted about AMR specifically in animals, the large majority referred to antibiotic use in farming:

I'm sure there was something on the BBC news about pigs, I'm sure there was pig farming because I was just tuning in and out of it. I wondered why they had pigs and then they had people in a hospital bed and I was getting them confused. I know it was something about pigs being prescribed antibiotics and that is somehow having a knock on effect for us [laughter] but the steps on that chain I'm not sure about. (*Rosemary, Dog owner*)

This lack of awareness of AMR in pets, let alone interspecies transmission risks, implied that it was highly unlikely that pet owners would be able to broach the topic with vets, take AMR into account in the treatment of their pets, and participate in decision making concerning antibiotics. These perspectives indicate that AMR literacy and scaffolding of antibiotic decision making may be important for pet owners.

Another challenge for pet-owners was the long term and collective nature of AMR which was not easily related to their own immediate predicament with their pet in the present:

I suppose with any of these future problems, problems are easily described as being something that’ll affect us in the distant future, there’s an issue with knowing how serious a risk is this, how much needs to be done *now* (*Dave, Dog Owner*)

As in this example, for most pet owners, AMR was perceived as distant, future problem, with many ambiguous and unknown dimensions. Some participants contrasted the seemingly distant threat of AMR with the immediacy of their pets’ health, which was more directly experienced as part of their everyday lives, and therefore of a bigger influence on decision-making processes about antibiotics. AMR was of such negligible concern, due to its abstract, complex and temporally distant nature, that it did not influence decisions about the immediate concern of pet health and wellbeing. In contrast, the suffering of the animal that many considered to be “part of the family” was a visceral, potently experienced phenomenon that pushed individuals to make financial and other sacrifices to ensure their pet got better.

**Fig. 1:**
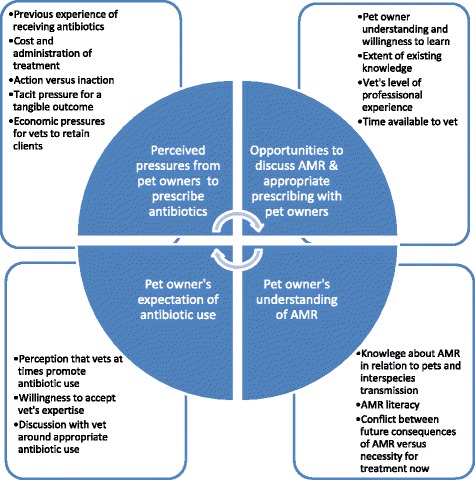
Factors shaping vet and pet owner interactions in relation to antibiotic prescribing and use

Figure 1 summarises results influences on pet owner and vet behaviours.

## Discussion

This paper is the first of its kind to examine *how* vets and pet-owners experience antibiotic decision-making, and identifying their subjective views on the factors which influence effective stewardship. This novel and inductive approach makes a valuable contribution by identifying the antecedents which underpin antibiotic prescribing behaviour (in vets) and usage (in pet owners). As such, it identifies several key ingredients for future intervention development.

First, it is important to acknowledge the interaction between pet owners and vets in relation to behaviours which might drive AMR. Vets appeared focussed upon the prescribing of antibiotics. They attributed over-prescribing to the complexity of the following drivers; perceived client satisfaction, commercial pressures to retain clients, the symbolic value of giving treatment, the influence of senior vets within practice, under-developed communication and persuasion skills, pet owners partial knowledge of appropriate antibiotic use and their very poor knowledge of antimicrobial resistance and interspecies transmission. Second, we must consider the nature of the interaction between pet owners and vets which may in itself facilitate AMR. For example, in the current study, pet owners had very poor knowledge and understanding of AMR and inter-species transmission but were aware of the negative consequences of inappropriate antibiotic use. They considered vets as being fundamentally responsible for prescribing decisions (attributing responsibility to their professional expertise). Pet owners did not report having high expectations of receiving antibiotic prescription. Finally, several key points are illuminated in terms of antimicrobial stewardship. Dissonance between the pet owner and vet appears to exist between their perceptions of where responsibility should rest in relation to the decision to prescribe antibiotics. Low levels of knowledge and understanding of AMR and its drivers amongst pet owners are important to note in this regard as they have the potential to constrain antimicrobial stewardship.

The apparent dissonance in perceptions of responsibility for antibiotic decision making for pets should be factored into effective stewardship interventions. Alternatively, vets and pet-owners only notice differing prescribing decisions which conflict with their a priori expectations (i.e. vets notice pressure to prescribe from pet-owners; pet-owners notice what they see as over-prescription). This possible explanation for dissonance implies that future work should focus on sensitising vets and owners to these situations. Dissonance may also result from a breakdown in communication, that is, misinterpretation of behavioural cues, mistaken inferences and so on, undertaken in the challenging context of time limited consultations with a pet-owner and their pet. The facilitation of more effective discussions between pet owner and vets around antibiotics might therefore be useful. Interventions should include professional development for vets, particularly those new to practice, to specifically facilitate more effective discussion around antibiotics. This may enable a more accurate identification and mitigation of pet owner pressures to prescribe. Potentially, standardised guidelines, vignettes and protocols could help pet owners and vets manage their interactions more effectively. Concurrent to the development of guidelines, there is potential to develop mandatory legislation, however this is contingent on political will and identifying suitable bodies to legislate and enforce. Specific training and guidelines with step-by-step protocols for engaging in conversations about antibiotic prescription have the potential to assist professionals and their clients ensure better decisions on antibiotics [19, 20]. As our research highlights, however, any attempt to shift the behaviours of vets will need support from clients, so interventions for vets should be run in parallel with complementary ones for owners.

Raising awareness and knowledge of AMR in pet owners themselves could be key to facilitating more productive interactions and discussions with vets around appropriate antibiotic use for their pets. Increased emphasis on appropriate, responsible stewardship could be especially effective in countering potential vet pressures to overprescribe, as pet owners might be more likely to resist with better knowledge resources. If negative consequences of over prescription were framed in terms of side effects for the animal (lessened future efficacy of antibiotics) it might be particularly effective. In this regard, increasing the self-efficacy of pet owners by teaching skills to participate in consultations might be useful. For example, vets claimed pet owners were unsatisfied if they did not receive antibiotics as they did not get “their money’s worth”. If pet owners’ awareness of the vets’ diagnostic processes was improved and therefore understanding of the informed decisions vets make was increased, potentially they would be more likely to perceive vet’s expertise as a reasonable outcome for the money they have spent.

There are some possible barriers to enhancing antibiotic decision-making, however. Research in other areas has shown that awareness alone is not sufficient to change individual behaviours [21], particularly for issues, like AMR, that are highly complex and seen to be a distant ambiguous risk. It is likely, therefore, that enhanced regulation of antibiotic prescribing in animal health will also be of value. In addition, standardisation and regulation of prescribing could be met with resistance by vets due to perceived to impositions on their autonomy and business models [22]. In addition, the governance of veterinary practice in the UK is fragmented into many different bodies, so cross practice guidelines would be challenging to develop and enact.

It is important to consider the key strengths and limitations of the current study. First, this paper presents a novel piece of work, original in its inductive, qualitative approach and innovative in its dual-focus (considering both the vet and pet-owners perspectives). This study contributes a rich, insightful understanding of AMR enabling behaviours from the perspectives of the participants themselves. It promotes increased awareness of the key drivers for antibiotic use/prescription, including underlying cognitive processes such as decision making and dissonance.

Various limitations have been identified for this study, primarily sources of bias within samples. The pet-owner sample were predominantly from middle-class, well-educated backgrounds (due to relatively poor success of other recruitment approaches). Vets who were recruited were likely to be already interested in AMR, and therefore potentially biased towards stewardship. Vets may have also been reserved in their disclosure due to a desire to project professionalism. The sample size reflects accepted standards for exploratory, theory-building qualitative health research. However, a larger sample could have alleviated some of the issues. The next step in exploring this topic would be a quantitative design informed by this research exploring behaviours of pet owners and vets regarding antibiotics use.

## Conclusions

This paper provides original evidence of the power of interactions between vets and pet owners to influence appropriate antibiotic prescribing and use in companion animals. In the absence of open communication, both vets and pet owners are inclined to misinterpret the intentions of the other, potentially leading to unnecessary prescription and inappropriate use of antibiotics.

Targeted multi-modal strategies which increase knowledge and awareness of antimicrobial resistance and appropriate antibiotic use in the pet owning public are important. These would provide a strong foundation on which effective interactions between vets and pet owners can be built, thus enhancing antibiotic stewardship behaviours. In addition, adequate time within consultations is required to enable vets to provide relevant information and to educate per owners around alternatives to antibiotic use.

By tackling the barriers of potential misunderstandings, lack of awareness of AMR, and lack of opportunity or capacity to discuss AMR during consultations, whilst enhancing the enablers of skilled communication which focuses on best clinical judgement as a precursor to client satisfaction, more effective antimicrobial stewardship can be achieved.

## Abbreviations

AMR: Antimicrobial Resistance
CARS: Control of Antimicrobial Resistance in Scotland
CVMP: Committee for Medicinal Products for Veterinary Use
HPS: Health Protection Scotland
